# Clinical outcomes following polymer-free sirolimus-eluting stent implantations in unselected patients: A descriptive subgroup analysis in patients with renal impairment

**DOI:** 10.1097/MD.0000000000021244

**Published:** 2020-07-17

**Authors:** Florian Krackhardt, Matthias W. Waliszewski, Behrouz Kherad, Claudia Barth, Daniele Marcelli

**Affiliations:** a Charité Universitätsmedizin, Internal Medicine and Cardiology, Campus Virchow; b Department of Medical Scientific Affairs, Vascular Systems, Aesculap, B. Braun Melsungen AG, Berlin; c Department of Medical Scientific Affairs, Avitum, B.Braun Melsungen AG, Melsungen, Germany.

**Keywords:** chronic kidney disease, dialysis dependence, polymer-free, sirolimus-eluting stent

## Abstract

Patients with chronic kidney disease (CKD are frequently excluded from coronary artery disease trials. The aim of this assessment was to study the clinical outcomes of polymer-free sirolimus-eluting stent implantations in patients with impaired renal function.

Large-scale, international, single-armed, multicenter, ‘all comers’ observational studies (ClinicalTrials.gov Identifier: NCT02629575 and NCT02905214) were used for this post-hoc subgroup analysis to compare the clinical outcomes in patients with normal renal function (NRF) to those with renal insufficiency (CKD, dialysis dependence). The accumulated target lesion revascularization rate was the primary endpoint at 9 to 12 months whereas the accumulated major adverse cardiac event, stent thrombosis (ST) and procedural success rates were part of the secondary endpoints.

There were 6791 patients with NRF, whereas 369 patients had CKD and 83 patients were dialysis dependent. The target lesion revascularization rate at 9 to 12 months was significantly higher in dialysis patients (2.1% vs 3.3% vs 6.7%, *P* = .011). The accumulated major adverse cardiac events rates in the dialysis and in the CKD group were significantly higher as compared to patients with NRF (13.3% vs 4.0%, *P* < .001; 6.5% vs 4.0%, *P* = .024). Finally, ST rates (NRF: 0.7%, CKD: 0.6%, dialysis: 1.3%) were not statistically different between subgroups (*P* = .768). All-cause cumulative mortality rates were 3.3% (CKD) and 4.0% (dialysis) respectively.

Percutaneous coronary interventions with polymer-free, ultra-thin strut sirolimus-eluting stents have comparable revascularization rates in CKD and dialysis dependent patients as compared to percutaneous coronary interventions with other 2nd generation drug-eluting stents. ST and all-cause mortality rates were low as compared to available literature references.

## 1 Introduction

Cardiovascular disease (CVD) and specifically coronary artery disease (CAD) constitute the leading cause of morbidity and mortality in patients with chronic kidney disease (CKD).^[1]^ The frequency for cardiovascular morbidity and mortality also increases with the degree of renal impairment.^[2]^ CVD in dialysis patients is responsible for approximately 45% of all-cause mortality.^[2]^ About one third of hospital admissions of CKD patients is due to CVD^[3]^ and many patients with CKD do not reach dialysis initiation^[2]^ due to cardiovascular events. According to the latest data reported by the US Renal Data System,^[3]^ 2,450,740 patients were treated worldwide for end stage renal disease (ESRD) in 2015. Half of the patients admitted to dialysis are already affected by CAD. The first month following dialysis initiation is characterized by a very high cardiovascular risk with cardiovascular event rates 3- to 8-fold higher than during the second year of dialysis.^[4]^ The increased burden of CVD in the CKD and dialysis population may be attributable to the higher prevalence of both, traditional risk factors as well as uremia-related and uremia-induced risk factors.^[5–7]^ Patients on dialysis are also characterized by an accelerated process of atherosclerosis^[1]^ and according to Eckardt et al,^[7]^ the coronary, cerebral, and peripheral arterial vasculatures are all affected.

There several treatment strategies described in the recent literature^[8]^ to treat stable CAD in CKD patients. Farkouh and coworkers investigated the optimal coronary revascularization strategy in patients with CKD and type 2 diabetes mellitus by using pooled patient level data. Their study revealed that in diabetic stable CAD patients, the combination of optimal medical treatment (OMT) and coronary artery bypass grafting (CABG) lead to similar 12-month revascularization rates of 12% to 14% as compared to OMT and percutaneous coronary intervention (PCI).^[8]^

PCI as one treatment option for renally impaired CAD patients has been studied with 2nd generation drug-eluting stent (DES) by Lee et al,^[9]^ who reported 1-year TLR rates of 2.7% in normal renal function (NRF) and 4.4% in CKD patients. Initiated by the findings of the COURAGE trial in stable CAD patients,^[10]^ OMT has received considerable attention and may be considered as a conjunctive treatment for PCI or CABG in patients without acute coronary syndrome (ACS).

As described in the literature it cannot be excluded that CKD patients may receive less aggressive cardio-protective therapy.^[11]^ Recently, new stent coating technologies using bioabsorbable polymers or non-polymer coating have been developed aiming to shorten dual antiplatelet therapy with the hypothesized reduced risk of bleeding due to a lower thrombogenic stimulus.^[12]^ Patients on hemodialysis with high risk of hemoconcentration and blood volume reduction during treatment and more compromised coronary arteries, may gain a higher benefit from a polymer-free sirolimus eluting stent (PF-SES)^[13–16]^ as compared to bare metal stents (BMS)^[17]^ and earlier DES generations. This would help to reduce the negative gap in terms of clinical outcomes currently present between patients with CKD, those on dialysis as compared to patients with NRF.

Therefore, considering the paucity of data currently available in patients with CKD and those on dialysis, we studied the clinical outcomes after PF-SES implantations in CKD and dialysis patients and compared these to results in patients with NRF. The aim of this study was to compare the rates of target lesion revascularization (TLR), major adverse cardiac events (MACE), stent thrombosis (ST) and mortality for comparison with the pertinent literature.

## 2 Materials and methods

Details on the study database were previously published by Krackhardt et al^[13–16]^ Briefly, the ISAR 2000 all-comers registry (ClinicalTrials.gov Identifier NCT02629575 and NCT02905214), prospectively enrolled patients in 43 European and 39 Asian cardiac centers. All relevant ethics committee votes were obtained prior to patient recruitment. For the French centers national approval was obtained by the Comité Consultative sur le Traitement de l’Information en matière de Recherche dans le domaine de la Santé (CCTIRS dossier no. 14.613) and the Commission Nationale de l’informatique et des Libertés (CNIL, demande d’autorisation no. 915019).

### 2.1 Endpoints

The accumulated TLR rate within a follow-up window of 9 to 12 months was the primary endpoint, whereas secondary endpoints were the MACE rate, the in-hospital MACE rate and the corresponding in-hospital rates of myocardial infarction (MI) and TLR (CABG and re-PCI). MI (in-hospital and cumulative) were defined by the 3rd general definition.^[17]^ The criteria for renal insufficiency and mandatory dialysis were glomerular filtration rate < 90 mL/min/1.73m^2^ and a cutoff glomerular filtration rate rate < 15 mL/min/1.73m^2^ respectively.

To define acute/subacute stent thromboses (ST) the academic research consortium criteria^[18]^ were used. The angulation criterion of >45° as described by Turgut et al^[19]^ defined severe target lesion tortuosity.

### 2.2 Devices

A PF-SES (Coroflex^©^ ISAR, Coroflex^©^ ISAR Neo B.Braun Melsungen AG, Germany) previously studied by Krackhardt et al^[13–16]^ was used in this study. Its sirolimus matrix coating was extensively investigated in the ISAR-TEST 5 trial with very favorable clinical outcomes up to 5 years.^[20]^ Briefly, the polymer-free matrix consisting of sirolimus and probucol is on the abluminal stent surface of an ultra-thin strut cobalt-chromium backbone. PF-SES were implanted in single or multi-vessel disease patients (≥18 years) with objective proof of ischemia with either stable angina or ACS. De novo and restenotic target lesions with reference vessel diameters from 2.0 to 4.0 mm were treated according to generally accepted recommendations.^[21]^

### 2.3 Procedures and co-medication

Due to the all-comers approach of this assessment, femoral or radial vascular access was permitted (≥5 French introducer). Direct stenting or pre-dilation with a balloon catheter of the center's preference could be chosen within the clinical routines in the participating study centers. All patients received intravenous heparin (70 IU/kg) prior to the procedure. Also prior to the procedure platelet aggregation inhibitor loading was recommended but not mandatory.

Depending on the clinical presentation of the patient, either clopidogrel 75 mg/day, prasugrel 10 mg/d or ticagrelor 2 x 90 mg/d were advised. Acetylsalicylic acid 100 to 325 mg/d was prescribed lifelong. Anti-coagulation therapy due to other concomitant conditions were maintained according to the patient's individual benefit/risk ratio for bleeding episodes.

For this large scale, unselected cohort, a dedicated and well documented^[13]^ electronic data capture system was utilized.

### 2.4 Statistics

This is a post-hoc descriptive analysis of patient groups with different degrees of CKD as compared to patients who are not renally impaired. Therefore, a samples size estimation was not conducted. Continuous variables were evaluated by 1-way Analysis of Variance and subgroup post-hoc multiple comparisons were performed by using the Tukey test. Dichotomous variables were analyzed by the 2-sided Fisher exact test or the Chi^2^ statistic whenever applicable.

Univariate Kaplan–Meier analysis was conducted to explore the association of chronic renal failure or the status of dialysis dependent with the development of MACE during the follow-up. Survival differences between groups were assessed using the log-rank test. Multivariate Cox proportional-hazard models adjusted for age, gender, diabetes, hypertension and coronary lesion characteristics assessed the association of presence of CKD or the status of dialysis dependence with the onset of MACE.

A *P*-value .05 was considered significant. SPSS version 24.0 (IBM, Munich, Germany) was used for all analyses.

## 3 Results

A total of 7243 patients were enrolled (Table 1). At baseline, 93.8% of patients had NRF, 5.1% had some degree of CKD and 1.1% were on dialysis (82 hemodialysis, 1 peritoneal dialysis). In relation to the overall population by region, the percentage of patients with CKD was higher in Europe (6.0% vs 2.0% in Asia) and of dialyzed patients was higher in Asia (2.5% vs 0.8% in Europe. Patients with CKD (72.8 ±10.6 years) were significantly older than those patients with NRF (66.0 ± 11.3 years) as well as those on dialysis (67.3 ± 10.2 years). No significant differences in terms of gender distribution were detected. The proportion of diabetics was significantly higher (*P* < .001) in patients with CKD (59.6%) and on dialysis (72.3%) as compared to patients with NRF (35.6%).

**Table 1:**
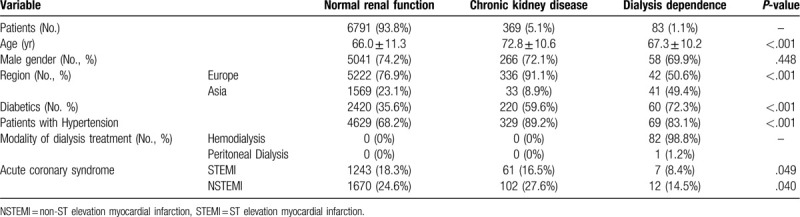
Baseline patient data.

NRF and CKD patients (Table 1) had a higher prevalence ACS as compared to the dialysis group (42.9% vs 22.9%, *P* < .001; 44.1% vs 22.9%, *P* < .001). The rates for ST elevation myocardial infarction (STEMI) and non- STEMI were also lowest in dialyzed patients (STEMI: 18.3% vs 16.5% vs 8.4%, *P* = .049; non- STEMI: 24.6% vs 27.6% vs 14.5%, *P* = .040).

Lesion characteristics of the 3 groups are reported in Table 2. In general, coronary arteries of CKD and dialysis dependent patients were significantly more compromised than in the NRF subgroup. With the exception of thrombotic occlusions, all other characteristics were significantly more frequent in the CKD or in the dialysis groups (ie, the frequencies of treated in-stent restenosis was NRF 2.9%, CKD 5.1%, dialysis 8.4%, *P* < .001 and of diffuse vessel disease was NRF 38.6%, CKD 51.5%, dialysis 48.4%, *P* < .001). There were more stents per patient used in patients with CKD and dialysis patients in comparison to those with NRF (NRF 1.25+/-0.63, CKD 1.33+/-0.78, dialysis: 1.33+/-0.73, *P* = .69).

**Table 2:**
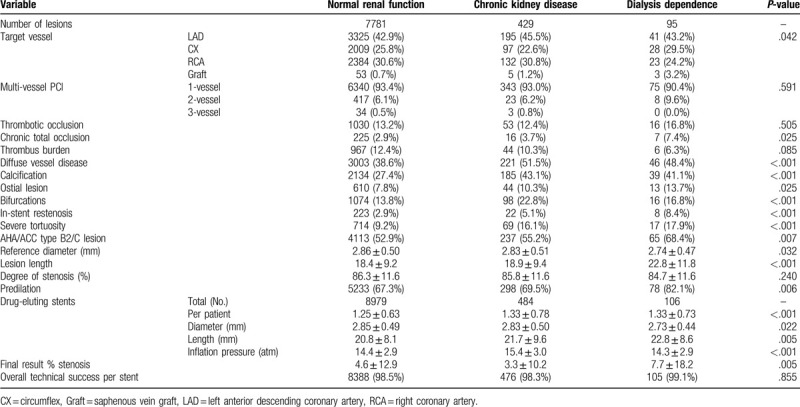
Lesion characteristics and procedural data.

**Figure 1:**
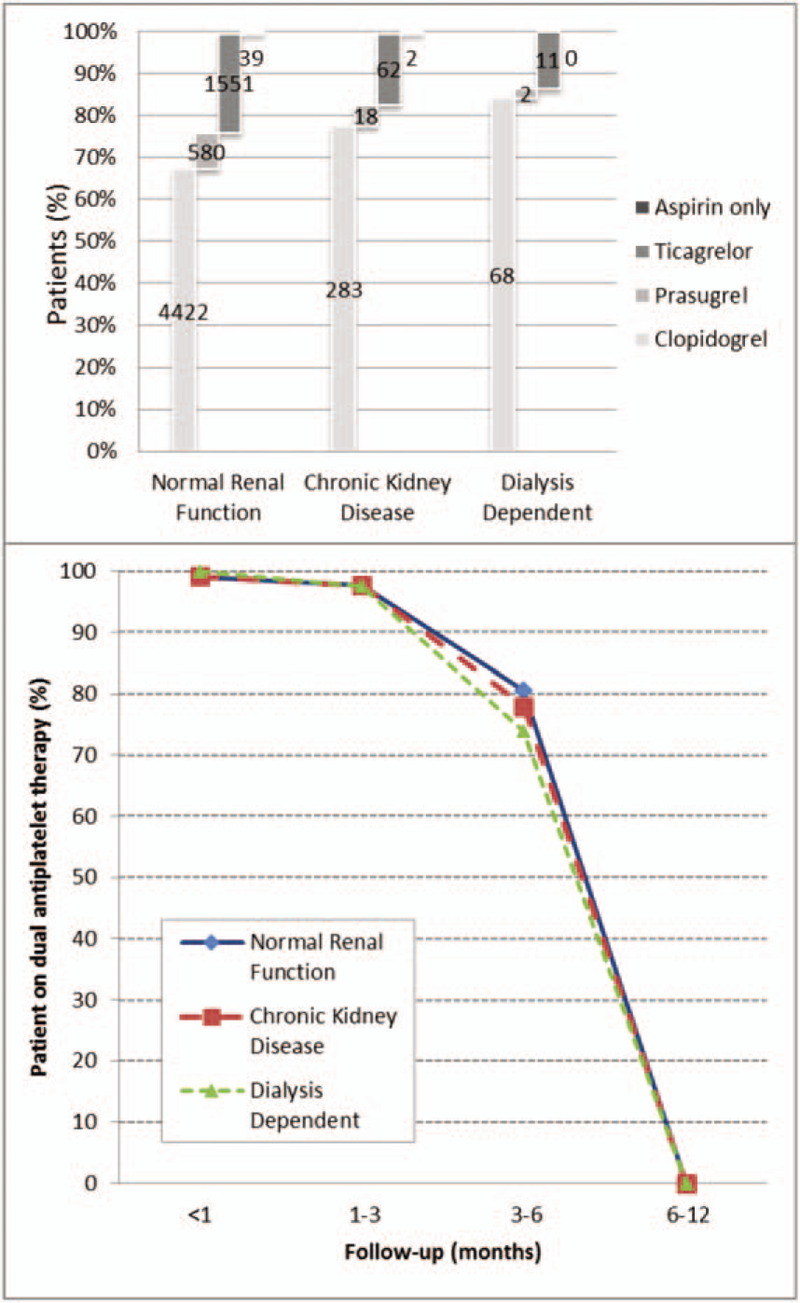
Distribution of post-procedural anti-thrombotic co-medication and their duration in patients with normal renal function, chronic kidney disease and dialysis dependent patients.

Peri-procedural co-medications prescribed before and after (Fig. 1) PF-SES implantations were similar. Differences relating to dual antiplatelet therapy during follow-up were also not detected.

The primary endpoint TLR at 9 to 12 months (Table 3) was significantly higher in dialysis patients (NRF 2.1%, CKD 3.3%, and dialysis 6.7%, *P* = .011). The cumulated MACE rate was significantly higher in patients with CKD and in those on dialysis (NRF 4.0%, CKD 6.5%, dialysis 13.3%, *P* < .001).

**Table 3:**
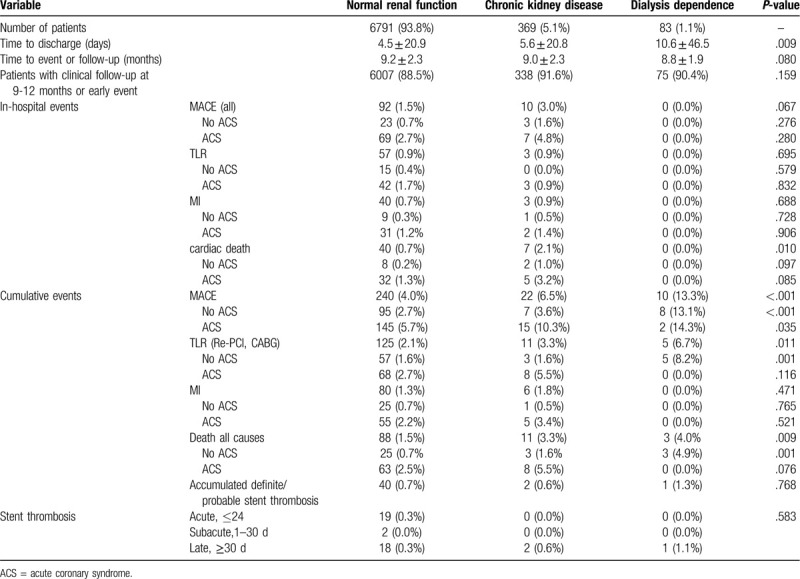
Clinical outcomes.

**Figure 2:**
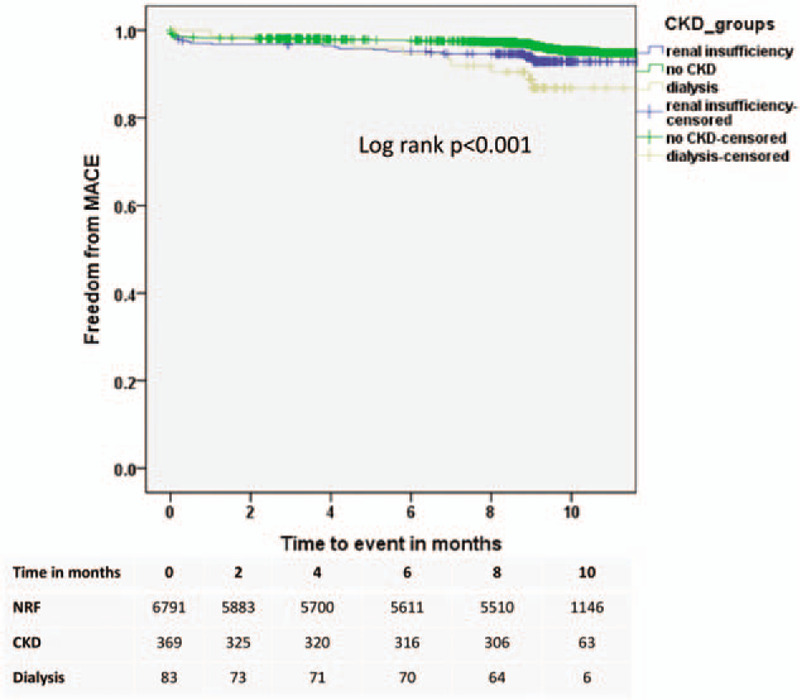
Kaplan-Meier analysis for freedom from major adverse cardiac events in patients with NRF, CKD and dialysis dependence.

**Figure 3:**
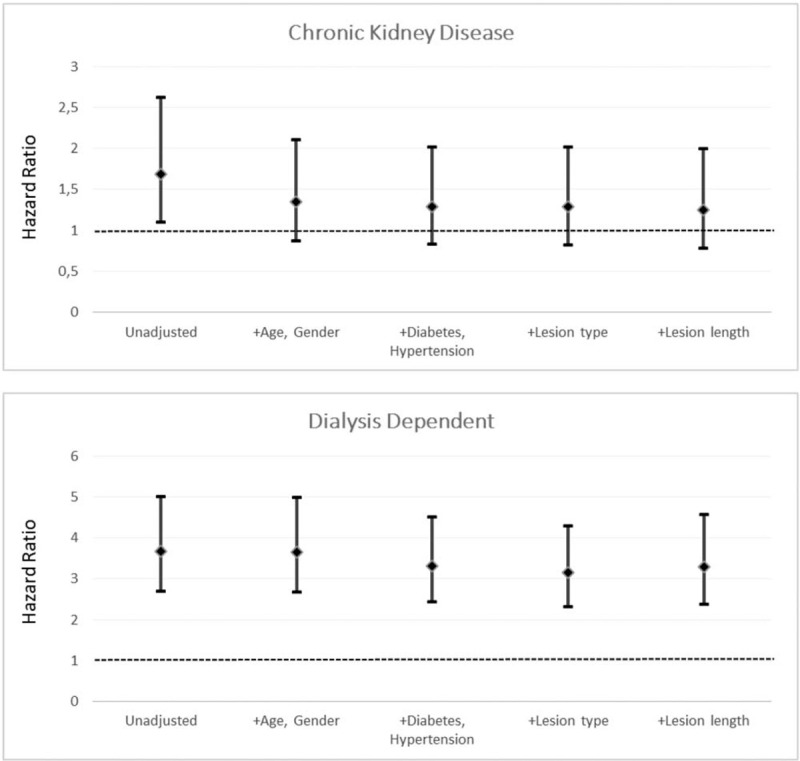
Hazard ratios in chronic kidney disease and dialysis dependent patients for selected demographic and lesion morphological factors.

Kaplan-Meier survival analysis (Fig. 2) revealed significant (log-rank test: *P* < .001) higher incidence of MACE in patients with CKD and in those on dialysis in respect to patients with NRF. When introducing in a stepwise pattern of demographic variables (age, gender), co-morbidities (diabetes, hypertension), and lesion morphological descriptions (lesion type, lesion length), the association with dialysis having a higher hazard ratio in respect to those NRF patients was maintained (Fig. 3). Older age was significantly associated with a higher hazard ratio for the development of MACE (per year: 1.04, 95% confidence interval 1.02–1.05; *P* = .010). In addition, the presence of diabetes was associated with a 28% borderline significant greater hazard ratio (HR 1.28, 95% confidence interval 0.98–1.67; *P* = .070).

All-cause mortality rates were significantly different between the NRF and CKD groups (1.5% vs 3.3%, *P* = .007) and borderline significant in comparison of NRF versus dialysis (1.5% vs 4.0%, *P* = .066). Furthermore, the accumulated ST rates were not significantly different between groups (NRF 0.7%, CKD 0.6%, dialysis 1.3%, *P* = NS) (Table 3). The rates of in-hospital events were not different between groups with the exclusion of cardiac death (NRF 0.7%, CKD 2.1%, dialysis 0.0%, *P* < .010). Intrahospital MACE was numerically higher in patients with CKD (NRF 1.5%, CKD 3.0%, dialysis 0.0%, *P* = .067).

## 4 Discussion

Due to the heterogeneity of studies in terms of follow-up duration, CKD definitions and comparators, it is methodologically challenging to compare our cumulative TLR, MACE, mortality and ST rates to those reported in the literature. However, a few milestone trials described important findings for a first orientation comparing CABG versus DES^[8,22,23]^ and interventional revascularization strategies with a number of different DES technologies.^[9,24]^ We found that the TLR rate in dialysis patients was 3-fold higher as the corresponding TLR rate in the NRF group and twice as high as compared to the CKD group. These increased revascularization rates have also been reported by Lee et al^[9]^ who reported similar outcomes in ESRF patients. Lee et al^[9]^ reported 1-year TLR rates of 2.7% in NRF and 4.4% in CKD patients which agree also well with our TLR rates of 2.1% (NRF) and 3.3% (CKD).

Farkouh and coworkers^[8]^ did not observe a significant difference in TLR rates at 12 months between OMT/CABG and OMT/PCI. They reported a 12-month TLR rate of 12% to 14% which is higher than our corresponding TLR rate. Most likely, this inter-study difference can be explained with their diabetes-only study population with a more pronounced multi-vessel disease state. Studies comparing CABG to DES implantations in CKD patients also focused on mortality as the primary endpoint. In terms of TLR, our dialysis dependent patient group had comparable outcomes (6.7%) to those after CABG (5.0%).^[22]^ Bangalore and coworkers^[23]^ compared CABG to everolimus-eluting stents (EES) and found a lower revascularization rate at 12 months in CKD patients who underwent CABG instead of EES implantations (5% vs 10%).

Roberts et al.^[22]^ did not observe any differences between CABG and DES in terms of their all-cause mortality or cumulative MACE rates. They reported 12-month mortality rates depending on the severity of CKD in the 10% to 20% range which are significantly higher than our all-cause mortality rates of 3.3% (CKD) and 4.0% (dialysis). Crimi et al^[24]^ investigated the use of EES, paclitaxel-eluting stents (PES), zotarolimus-eluting stents and bare-metal stents (BMS). They reported the lowest 2-year MACE rate of 26.4% in patients treated with EES. Despite our short follow-up of 9–12 months, our MACE rates ranged from 4.0% in NRF, 6.5% in CKD to 13.3% in dialysis patients, they seem to be in good agreement with the outcomes after zotarolimus-eluting stent implantations^[24]^ and comparable to the outcomes after CABG.^[22]^

We observed more complex lesion morphologies in the CKD and in the dialysis groups which were characterized with higher rates of calcification and diffuse vessel disease as compared to NRF vessels (Table 2). This is a common finding which was observed in a number of studies.^[25]^ The fact that the reference vessel diameters were also smaller in dialysis patients also agrees with a number of other study reports. In patients with CKD and even more in those on dialysis, 2 types of vascular calcifications can be described, arterial media calcification (calcific arteriosclerosis) and accelerated calcification of intimal plaques (calcific atherosclerosis).^[25]^ In the current study we used a polymer-free DES based on a pre-mounted, thin strut (50–60 mm) cobalt-chromium stent. Due to its flexibility and lesion crossability the study device had similarly high success rates (*P* = .855) in all subgroups, i.e. 99.1% in dialyzed (105/106) vs. 98.3% (476/484) in CKD patients vs 98.5% (8388/8979). Lee et al^[9]^ reported technical success rates >98% which agree well with our findings.

Our results show that the primary endpoint of the study, the accumulated TLR and MACE rates were significantly higher in dialysis patients as compared to patients with NRF or CKD. CKD patient were on average about 7 years older than those with NRF and also older than dialysis patients, which appears to be substantially important to skew the true difference in ischemic risk between CKD and dialysis patients.

Regarding ST, the cumulative event rate was low (Table 3: 0.7%–1.3%) and only the group of patients with NRF experienced acute (within the 24-hours) and subacute events (1 to 30 days). The cumulated event rates were not statistically different between groups. The study by Hassani et al^[26]^ in dialysis patients treated with SES or PES, revealed, a significantly higher rate of subacute ST (3.1% vs 0.3% in non-dialyzed patients, *P* < .001) without significantly different rates of late ST (0% vs 0.3%, *P* = NS). We did not observe higher ST rates in patients with renal impairment which may be due to the polymer-free stent surface and its more rapid stent strut coverage as observed in preclinical studies.^[27]^

Antiplatelet therapy has been previously reported to be associated with a lower rate of ischemic events and mortality in patients with CKD treated for ACS, but without increasing the risk of major bleeding.^[28]^ However, more recent findings of a higher bleeding risk associated with long term prescription has to be considered.^[29]^

## 5 Limitations

The main limitation of this study is its observational nature despite the prospectively collected data. Due to the observational statistics used, confounding factors may also have played an important role in our findings. Inherent to ‘all-comers’ studies within the framework of clinical routines, there is the potential to event underreporting. However, our overall follow-up rate of 88.6% fares well with other studies of this magnitude. The subgroups were not well balanced by nature (83 patients on dialysis, 369 patients with CKD, and 6791 patients with NRF) which is unfortunate for statistical power. However, it represents a ‘real life’ experience of percutaneous revascularization in renally impaired patients. This very high-risk population is, however, routinely excluded from randomized trials.^[30]^ It also would have been desirable to include data on ventricular function which presents a shortcoming of this study. Despite our high prevalence of diabetics in our study, the prevalence of multi-vessel disease was lower than normally expected.

## 6 Conclusions

Despite higher rates for MACE and TLR in CKD patients or those on dialysis, polymer-free SES implantation is a treatment option with comparable clinical rates as compared to other 2nd generation DES from other reports. ST and all-cause mortality rates were low as compared to available literature references.

## Acknowledgments

This research would not have been possible without the orchestrated network of clinical support provided by Denny Herberger (Germany), DR Ghislaine Martin, Ms. Aude Michaud and Ms. Lucie Wachowiak (France), DR Ricard Rosique and Marco Mantilla (Spain), Ms. Zoey Hooi (Malaysia) and Ms. Yoonmi Lee (South Korea). Furthermore, essential statistical support was provided by DR Ralf Degenhardt at the Cardiovascular Research Center in Rotenburg, Germany.

## Author contributions

All authors: conception and design of the study, writing the manuscript and interpretation of the data, statistical analysis DM, FK and MWW.
